# Dataset of motivational factors for using mobile health applications and systems

**DOI:** 10.1016/j.dib.2023.109589

**Published:** 2023-09-16

**Authors:** André Henriksen, David-Zacharie Issom, Ashenafi Zebene Woldaregay, Gerit Pfuhl, Eirik Årsand, Keiichi Sato, Gunnar Hartvigsen

**Affiliations:** a Department of Computer Science, UiT The Arctic University of Norway, Tromsø, Norway; b University Hospital of Geneva, Geneva, Switzerland; c Department of Psychology, UiT The Arctic University of Norway, Tromsø, Norway; d Department of Psychology, Norwegian University of Science and Technology; e IIT Institute of Design, Chicago, USA

**Keywords:** mHealth, Survey, Wearables, Sensors, Sharing, Integration, Social media, Mobile apps

## Abstract

We created and carried out a cross-sectional anonymous structured questionnaire on what motivates users of mobile health applications and wearables to share their collected health related data. The questionnaire was distributed online in English, French, and Norwegian. In addition, a flyer with information of where to locate the online questionnaire was distributed during a Swiss health conference. We used snowball sampling and encouraged participants to forward the questionnaires to friends, family, and others. Data were collected between October 2018 and March 2020. 58.1 % (*n* = 473) responded to the English survey, 34.3 % (*n* = 279) responded to the French survey, and 7.6 % (*n* = 62) responded to the Norwegian survey.

The questionnaire contained 38 questions divided into seven themes: Background and health goals, Wearables and sensors, Mobile applications, Logging of health data, Data sharing- and integration, Social media and entertainment, and Demographics (age, gender, country of origin, chronic disease status, and chronic disease caretaker status). Answer options were single answer, multiple-choice, open-ended, or on a 4-point Likert scale.

Questions were defined based on 16 in-person interviews with people without any chronic disorder, people with diabetes, and people with sickle cell disease. All questions were optional. Data were collected from 814 participants. All answers to the open-ended questions have been translated into English.

This dataset is especially interesting for researchers interesting in what motivates people with and without chronic disease across countries to use mHealth tools and share their collected health data. Only a subset of variables has been analyzed so far and new research questions on motivation can potentially be answered using this dataset.

Specifications Table

**Table:** 

Subject	Health informatics

Specific subject area	Motivational factors for mobile health (m-health) data sharing
Type of data	Table
How the data were acquired	Data collection was handled by questionnaires created with nettskjema.no, a survey solution developed and hosted by the University of Oslo (nettskjema@usit.uio.no). The survey questions were created based on 16 in-person interviews [1,2] and discussions among the authors. An English, French, and Norwegian version of the survey was distributed online. The survey was open between October 2018 and March 2020. A copy of the (English) survey is available in the data repository (Questionnaire.pdf).
Data format	Raw
Description of data collection	We aimed to collect survey data on healthy individuals, as well as people with chronic disease, especially people with diabetes and sickle cell disease. Inclusion criteria was being 18 years or older. 814 participants completed the survey. The survey contained 38 questions, grouped into seven themes.
Data source location	UiT The Arctic University of Norway, Tromsø, Norway
Data accessibility	Repository name: DataverseNO [3] Data identification number: doi:10.18710/AOQF05 Direct URL to data: https://dataverse.no/dataset.xhtml?persistentId=doi:10.18710/AOQF05

## 1 Value of the Data

• Motivation for using mobile health applications and systems is a big challenge in self-care and disease management. Offering our dataset to the research community might generate new knowledge that can increase usage of such apps and systems.
• Researchers interested in the background data and how findings may differ between disease groups can benefit from these data by stratifying data in new ways.
• Only a subset of the data in the dataset has been used in published articles. Researchers interested in motivation and mobile health can benefit from this data by creating their own research questions from the unused questions.
• These data add to the original publications as it enables further analysis and stratification on all demographic data (e.g., language and country). Analysis in original publications were limited to a few variables only.
• These data are also a timestamp to allow comparing them to newer, not yet collected data and the change in motivation to share health data.
• These data are useful because they allow other researchers to verify the findings in the original publications [4], [5], [6], and the data can be further analyzed for new research questions using the same variables.

## 2 Objective

The original survey was created to better understand what could motivate users of mHealth apps and wearable devices to share their collected data. Although the initial focus was on how healthy people responded differently from people with a chronic disorder, the dataset can be used beyond the original scope to answer research questions without this specific stratification.

The 38 included questions are grouped into seven themes (T1-T7): T1) Background and health goals, T2) Wearables and sensors, T3) Mobile applications, T4) Logging of health data, T5) Data sharing- and integration, T6) Social media and entertainment, and T7) Demographics (age, gender, country of origin, chronic disease status, and chronic disease caretaker status). The analysis of the data has so far focused on how motivation differs between healthy people and those with a chronic disease within some of these themes.

As of March 2023, three publications [4], [5], [6] have been published using this dataset. In these articles, only a subset of questions has been analyzed. Specifically, answers to questions 2 and 31 (Theme T1), 32-33 (Theme T3), 12 (Theme T4), 18 and 34-37 (Theme T5), as well as 20-24 (Theme T6), have not been analyzed. New research questions can potentially be identified and answered using the unused survey questions. Additionally, by conducting further comparisons and stratification or removing the stratification based on chronic disease status, new research questions can be identified using the already used survey questions.

## 3 Data Description

Data were collected from 814 participants anonymously. The dataset is available on DataverseNO [3]. Alongside a readme file (“00_ReadME.txt”) that provides information about the files within the dataset, there are three primary files: “*Questionnaire.pdf*”, “*Responses.csv*”, and “*Questions-metadata.csv*”.

The “*Questionnaire.pdf*” contains a complete list of the 38 questions included in the survey, along with the associated answer options. Additionally, the question type is given, stating whether the question is *single-answer, multiple-choice, open-ended*, or rated on a 1-4 *Likert scale*.

The “*Responses.csv*” contains the 814 responses to the survey, with one answer per row. The first row serves as the data header, where the value gives the Questions ID (1-140). More detailed information about each question can be found in the metadata file (*Questions-metadata.csv).* Multiple choice questions (Question NR 6, 11, 16, 19, and 22) have been split into distinct questions within this file. Additionally, questions that include an open-ended answer option (text) are treated as separate questions. As a result, the original set of 38 questions have been expanded to 140 unique questions. Within the dataset, the designation “NA” indicates that the participant did not provide an answer to the respective question.

The “*Questions-metadata.csv*” file contains information about the questions and each answer option.

Several questions (Question NR 3, 7, 9, 10, 14, 15, 17, 18, 23, 24, 37, 38) were presented to the participant as a matrix, where each answer option was displayed on a Likert scale. Although they were presented as a single question to the participant, they are stored as separate questions in the dataset. Consequently, several questions (represented by Question ID) are associated with the same overarching question (represented by Question NR). Refer to Table 1 for an overview of the column content within the “*Questions-metadata.csv” file*. The Question ID in this file corresponds to a linked question in the response file (*Responses.csv*).

**Table 1:** Content description of “Question-metadata.csv”.

Column	Description
1	Question ID: 1-140. 38 questions where multiple choice questions and open-ended answers are divided into separate questions. Totaling 140 unique questions.
2	Question NR: 1-38. 38 questions as presented to the participants.
3	Questions section. Questions were divided into the following sections: Background, Wearables and sensors, Mobile apps, Logging, Data sharing and integration, Social media and entertainment factors, and Optional questions
4	Questions text: The question text
5	Answer options: Available answer options for a question

Participants who answered “Yes” to Question NR 100 (Question ID 111) were asked nine additional questions. In the dataset, “NR 100” was used to emphasize this question and differentiate it from the rest of the questions.

In Woldaregay et al. [6] (2020), the authors reported on questions related to data sharing and data integration (Theme T2, Question 6, 16, 17, 19, 38). Responses were stratified by respondent disease status (Theme T7, Question 28). The analysis was conducted before the survey was closed. Therefore, only the first 447 respondents were included in the analysis for this article. The subset of data used in Woldaregay et al. has been published in a separate repository [7]. No data paper was written for this limited data repository. An additional 367 respondents completed the final survey. The aim of this article was to determine which factors could motivate people to share self-collected health data.

In Bradway et al. [4], the authors reported on question related to primary health goals and background (Theme T1, Question 1, 3, 4, and 30), health technology usage (Theme T2, Question 6), motivation for collecting health data (Theme T4, Question 13). Responses were limited to caregivers (*n* = 72) of individuals with chronic disease (Theme T7, Question 29) and stratified by their own chronic disease status (Theme T7, Question 28). No other questions have been analyzed for this respondent group. The aim of this article was to focus on the caretakers of people with chronic disease and determine whether they are motivated by mHealth technology for achieving their health goals.

In Henriksen et al. [5], the authors reported on questions related to wearables and sensors (Theme T2, Question, 6, 7, 8, and 9), use of mobile applications (Theme T3, Question 10 and 11), and data logging (Theme T4, Question 14 and 15), as well as questions related to using wearable devices (Theme T1, Question 5) and chronic disease status (Section S7, Question 28). All respondents (*n* = 814) were included in the analysis. The aim of this article was to identify factors that motivates people (with and without chronic disease) to use mobile health sensors and applications.

## 4 Experimental Design, Materials, and Methods

The survey comprises 38 questions organized into seven themes. The questions were created based on insights gained from three semi-structured interviews, where a total of 16 people were interviewed. One interview guide was specifically designed for participants (*n* = 10) with sickle cell disease and their caretakers. Results from this study has been published by Issom et al. [1]. Two additional interview guides were created targeting individuals with diabetes (*n* = 2) and individuals with no chronic disease (*n* = 4). Combined results from all three interviews were published by Woldaregay et al. [2]. Through author discussion, the findings from these interviews were used to construct the final questionnaire and the themes. The questionnaire underwent a pilot test with colleagues (not experts in the field) before its finalization.

The questions were translated to Norwegian, English, and French and distributed online as a cross-sectional survey. The questionnaires were created using Nettskjema.no, a survey solution developed and hosted by the University of Oslo. Responses to open ended questions were translated to English.

Information about the survey was distributed in English and French using flyers at the Planète Santé (Planet health) conference in Switzerland (October 4^th^ to 7^th^, 2018). In addition, the questionnaires were distributed in four English diabetes Facebook groups (“CGM in the cloud”, “Spike App”, “CGMitC Off Topic #T1DIY”, and “Nightscout for Medtronic”) and five Norwegian diabetes Facebook groups (“For oss med Diabetes”, “Diabetesforbundet”, “Sweet girls and sugar dadies”, “Nightschout Norge”, and “Freestyle Libre Norge”). We used snowball sampling and encouraged participants to forward the questionnaires to friends, family, and others. The survey was open between October 2018 and March 2020.

Of the 814 respondents, 58.1 % (*n* = 473) responded to the English survey, 34.3 % (*n* = 279) responded to the French survey, and 7.6 % (*n* = 62) responded to the Norwegian survey.

## Ethics statements

Data were collected anonymously. Participants consented by starting and completing the online survey. No human or animal experiments were conducted. The study was vetted by the Regional Committee for Medical Research Ethics Northern Norway, REK North (2017/562/REK nord). The Norwegian centre for Research Data (NSD) (54558/3/LB) reviewed the study protocol and recommended the project to proceed. NSD changed name to SIKT in 2022.

## CRediT author statement

**André Henriksen**: Conceptualization, Methodology, Investigation, Data collection, Data curation, Writing - Original Draft, Writing – Review & Edit, Project Administration **David-Zacharie Issom**: Conceptualization, Methodology, Investigation, Data collection, Writing – Review & Edit **Ashenafi Zebene Woldaregay**: Conceptualization, Methodology, Investigation, Data collection, Writing – Review & Edit **Gerit Pfuhl**: Conceptualization, Methodology, Investigation, Data collection, Writing – Review & Edit **Eirik Årsand**: Conceptualization, Methodology, Investigation, Data collection, Writing – Review & Edit **Keiichi Sato**: Conceptualization, Methodology, Investigation, Writing – Review & Edit **Gunnar Hartvigsen**: Conceptualization, Methodology, Investigation, Writing – Review & Edit, Supervision

## Data Availability

Replication Data for: Dataset of motivational factors for using mobile health applications and systems (Original data) (DataverseNo)
